# 肺癌患者淋巴细胞亚群在外周血中的表达及其与预后的关系

**DOI:** 10.3779/j.issn.1009-3419.2011.08.06

**Published:** 2011-08-20

**Authors:** 君 罗, 志强 凌, 伟敏 毛

**Affiliations:** 310022 杭州，浙江省肿瘤医院，浙江省肿瘤研究所 Zhejiang Cancer Hospital, Zhejiang Cancer Research Institute, Hangzhou 310022, China

**Keywords:** 肺肿瘤, 淋巴细胞亚群, 化疗, Lung neoplasms, Lymphocyte subsets, Chemotherapy

## Abstract

**背景与目的:**

肺癌是最常见的恶性肿瘤之一，本研究旨在探讨肺癌患者外周血中淋巴细胞亚群的表达及与预后的关系。

**方法:**

采用流式细胞仪检测221例原发性肺癌首诊患者外周血淋巴细胞亚群CD3^+^、CD4^+^、CD8^+^、CD4^+^/CD8^+^、CD19^+^、CD25^+^、CD44^+^及NK细胞所占比例，并与96例健康人的血标本对比，结合临床及随访资料进行统计分析。

**结果:**

与健康对照组对比，肺癌患者淋巴细胞亚群8项指标中CD3^+^及CD8^+^明显低于健康对照组，CD4^+^/CD8^+^、CD19^+^、CD25^+^、CD44^+^及NK细胞明显高于健康对照组(*P* < 0.05)。与非小细胞肺癌(non-small cell lung cancer, NSCLC)相比，小细胞肺癌(small cell lung cancer, SCLC)的CD8^+^明显升高而CD4^+^和CD4^+^/CD8^+^明显下降(*P* < 0.05)。化疗后与化疗前相比CD3^+^明显上升，NK细胞、CD19^+^及CD44^+^明显下降(*P* < 0.05)，其中CD44^+^在化疗后表达不升高者有生存优势(*P*=0.021)，而其余3项指标与患者预期生存无关。

**结论:**

肺癌患者外周血淋巴细胞亚群普遍发生改变，CD44^+^在化疗后的改变可能与预后相关。

## Abstract (en)

**Background and objective:**

Lung cancer is one of the most common malignancies. The aim of this study is to investigate the relationship between the change of lymphocyte subsets in the peripheral blood of lung cancer patients and the survival rate.

**Methods:**

Flow cytometry was used to measure the percentages of lymphocyte subsets (CD3^+^, CD4^+^, CD8^+^, CD4^+^/CD8^+^, CD19^+^, CD25^+^, CD44^+^, and NK cells) in peripheral blood obtained from 221 patients with primary lung cancer without any treatment and from 96 healthy blood donors as the control group. The result was combined with clinical and follow-up data and statistical analysis was conducted.

**Results:**

The levels of CD3^+^ and CD8^+^ in the patient group are significantly lower compared with the control group, whereas the levels of CD4^+^/CD8^+^, CD19^+^, CD25^+^, CD44^+^, and NK cells are significantly higher (*P* < 0.05). CD8^+^ is significantly higher in the small cell lung cancer (SCLC) group compared with the nonsmall cell lung cancer (NSCLC) group. However, CD4^+^ and CD4^+^/CD8^+^ are lower in SCLC (*P* < 0.05). There were no significant differences in different stages and differentiation (*P* > 0.05) in the NSCLC group. The level of CD3^+^ was significantly higher compared with the pre-chemotherapy group, but NK cell, CD19^+^, and CD44^+^ were distinctly lower in the post-chemotherapy group (*P* < 0.05). More survival opportunities will be obtained for patients with no increase in CD44^+^ after chemotherapy (*P*=0.021), but the other three indices have no obvious influence on survival.

**Conclusions:**

Widespread changes of lymphocyte occur in the peripheral blood of patients with lung cancer. There is a significant correlation between the change of CD44^+^ and the prognosis after chemotherapy.

肺癌是最常见的恶性肿瘤之一，5年生存率仅有10%-15%。近年研究发现肺癌常伴有免疫机能紊乱，许多研究^[1-4]^报道了免疫细胞亚群与临床病理学资料之间的关系，却鲜见外周血免疫学指标与预后关系的阐述。本研究采用流式细胞技术，根据白细胞表面抗原分化群(clusters of differentiation, CD)对淋巴细胞亚群的分类来检测肺癌首诊患者外周血中的CD3^+^、CD4^+^、CD8^+^、CD4^+^/CD8^+^、CD19^+^、CD25^+^、CD44^+^及NK细胞，以正常参考值做对照，结合临床病理及随访资料进行分析，旨在探讨淋巴细胞亚群与患者预后的关系。

## 1 材料与方法

### 1.1 材料

收集2006年8月-2007年12月在浙江省肿瘤医院住院治疗的221例肺癌首诊患者资料，所有研究病例均经纤维支气管镜或肿块穿刺活检明确诊断为肺癌，并且抽血化验前均未行放疗、化疗或手术，排除严重感染性疾病或自身免疫性疾病患者。年龄33岁-83岁，男性175例，女性46例，中位年龄59岁。181例患者接受了含铂类两药联合化疗，其中78例患者在化疗后3周-4周再次抽血复查。96例健康成年人作为对照组，选自本院体检健康人群，年龄性别均与检测组匹配。对全部肺癌患者进行电话随访，随访截止日期为2010年12月10日，其中失访18例，失访者在生存分析中以最近一次随访日期作为截尾值处理。

### 1.2 肿瘤分期及分化程度

肿瘤分期根据纤维支气管镜、胸部C T、B超等检查结果，采用国际抗癌联盟(international union against cancer, UICC)2002年肺癌第六版的TNM分期标准，其中非小细胞肺癌(non-small cell lung cancer, NSCLC)188例：包括Ⅰ期8例、Ⅱ期7例、Ⅲ期80例、Ⅳ期84例、无分期资料9例；小细胞肺癌(small cell lung cancer, SCLC)33例。低分化、未分化124例，高分化、中分化及高中分化24例，分化程度未知73例。

### 1.3 淋巴细胞亚群检测

#### 1.3.1 标本采集

221例患者和96例健康对照组于入院治疗或体检之前清晨空腹抽静脉血2 mL，乙二胺四乙酸(ethylene diamine tetraacetic acid, EDTA)钾盐抗凝。获得标本后立即送检。

#### 1.3.2 试剂和仪器

采用美国Becton Dickinson公司FACS Calibur流式细胞仪和配套的淋巴细胞亚群检测试剂盒(Smultest IMK-Lymphocyte)，试剂盒中的双色荧光抗体为异硫氰酸荧光素和藻红蛋白标记的鼠抗人单克隆抗体(catalog No.340182)。

#### 1.3.3 三色免疫荧光染色

采集血液2 mL，肝素抗凝，2 h内送达实验室。流式试管内加相应单抗20 μL，加入肝素抗凝的待测血浆100 μL，混匀后室温避光30 min充分反应。加2 mL溶血素2次，震荡混匀，室温避光5 min，1, 200 rpm离心5 min，去上清后加磷酸盐缓冲液震荡混匀，1, 200 rpm离心5 min，倒去上清，加固定液300 μL震荡混匀。

#### 1.3.4 流式细胞仪分析

使用Couter公司配套的System Ⅱ系统软件数据处理系统。进行光路流路的较准后再进行荧光补偿，最后行样本检测。

### 1.4 统计学分析

检测结果以Mean±SD表示。利用SPSS 17.0分析试验结果，采用独立样本*t*检验、配对*t*检验、卡方检验、生存分析等方法分析数据。以*P* < 0.05为差异有统计学意义。

## 2 结果

### 2.1 临床病例资料分析结果

与健康对照组相比，肺癌患者淋巴细胞亚群除CD4^+^外的7项指标均有统计学差异，其中CD3^+^及CD8^+^明显低于健康对照组，CD4^+^/CD8^+^比值、NK细胞、CD19^+^、CD25^+^及CD44^+^明显高于健康对照组(表 1)。与NSCLC相比，SCLC的CD8^+^明显升高而CD4^+^和CD4^+^/CD8^+^比值明显下降(*P* < 0.05)。NSCLC中Ⅰ/ Ⅱ期与Ⅲ/Ⅳ期相比、肺癌患者低/未分化与中/高分化相比，淋巴细胞亚群的8项指标均无统计学差异(*P* > 0.05) (表 2)。

**1:** 肺癌患者与健康人外周血淋巴细胞亚群的对比 Contrast of lymphocyte subsets in peripheral blood between lung cancer patients and healthy control group

Lymphocyte subsets	Patients (*n* =221)	Healthy control group (*n* =96)	*P*
CD3^+^	66.20±12.26	73.32±7.65	< 0.01
CD4^+^	39.57±11.15	39.74±6.51	0.82
CD8^+^	23.17±9.08	28.68±4.83	< 0.01
CD4^+^/CD8^+^	2.08±1.31	1.44±0.42	< 0.01
NK	20.43±11.53	17.10±8.00	< 0.01
CD19^+^	8.56±4.55	7.91±2.98	0.03
CD25^+^	28.40±9.79	17.10±5.21	< 0.01
CD44^+^	66.37±20.23	45.81±13.50	< 0.01

**2:** 肺癌患者外周血淋巴细胞亚群与临床病理学特征的关系 Relationship between lymphocyte subsets in peripheral blood and clinicopathologic

Lymphocyte subsets	NSCLC clinical stage(cTNM)			Differentiation			NSCLC	
	Ⅰ/Ⅱ (*n* =15)	Ⅲ/Ⅳ (*n* =164)		High/Moderate (*n* =24)	Poor (*n* =124)		Yes (*n* =188)	No (*n* =33)
CD3^+^	68.87±12.04	65.81±12.43		68.29±11.82	65.99±11.89		64.91±12.09	66.43±12.31
CD4^+^	39.93±11.18	38.44±10.71		41.17±9.45	39.78±11.26		43.39±12.60^*^	38.90±10.77^*^
CD8^+^	24.73±13.33	24.05±8.98		24.75±11.52	22.97±8.58		18.03±6.27^*^	24.07±9.21^*^
CD4^+^/CD8^+^	2.07±1.18	1.91±1.12		2.04±1.10	2.10±1.39		2.89±1.90^*^	1.94±1.13^*^
NK	19.73±11.62	21.11±11.82		19.33±11.33	20.61±11.45		18.82±10.78	20.71±11.66
CD19^+^	6.40±3.36	8.64±4.84		9.04±6.13	8.29±4.33		9.48±3.68	8.39±4.67
CD25^+^	28.73±9.87	27.56±9.69		31.38±12.30	28.80±9.18		30.09±9.29	28.10±9.87
CD44^+^	62.93±23.76	66.91±19.99		64.17±19.61	66.69±19.38		65.48±20.13	66.52±20.29
^*^P < 0.05; NSCLC: non-small cell lung cancer.								

### 2.2 化疗相关的随访资料分析结果

化疗前后相比，肺癌患者淋巴细胞亚群中CD3^+^、CD19^+^、CD44^+^及NK细胞这4项指标具有统计学差异(*P* < 0.05)，其中化疗后CD3^+^明显上升，NK细胞、CD19^+^及CD44^+^明显下降(表 3)。化疗后CD44^+^升高组29人，未升高组49人，*Kaplan-Meier*分析提示化疗后CD44^+^表达不升高者有生存优势(*P*=0.021)，而其余3项指标与患者预期生存无关(*P* > 0.05)(表 4，图 1)。化疗后CD44^+^升高组(69.45 ±17.89)与未升高组(46.45±18.33)相比有统计学差异(*P*=0.01)，但两组的性别、年龄、吸烟史、饮酒史和家族史相比无统计学差异。*COX*回归分析显示化疗后CD44^+^表达不升高者有独立的生存优势(RR=1.863, 95%CI: 1.063-3.261, *P*=0.03)，其余3项指标与患者预期生存无关(*P* > 0.05)。

**3:** 肺癌患者外周血淋巴细胞亚群在化疗前后的对比(*n* =78) Contrast of lymphocyte subsets in peripheral blood between pre-chemotherapy and post-chemotherapy (*n* =78)

Lymphocyte subsets	Pre-chemotherapy	Post-chemotherapy	*P*
CD3^+^	66.40±12.59	71.58±12.44	< 0.01
CD4^+^	39.77±11.62	41.85±13.72	0.13
CD8^+^	23.33±9.22	25.24±11.15	0.11
CD4^+^/CD8^+^	2.11±1.39	2.27±2.02	0.40
NK	19.14±11.91	16.35±9.70	< 0.01
CD19^+^	9.06±4.49	6.10±4.56	< 0.01
CD25^+^	28.76±8.59	30.36±12.45	0.19
CD44^+^	66.94±19.41	55.00±21.24	< 0.01

**4:** 化疗后上升组与未上升组的生存期对比 Contrast of survival times between increase group and un-increase group after chemotherapy

Lymphocyte subsets	Average of survival times(months)		*P*
	Increase group after chemotherapy	Un-increase group after chemotherapy	
CD3^+^	21.14±2.12	22.20±3.49	0.69
NK	21.22±3.36	21.67±2.19	0.91
CD19^+^	21.71±4.01	21.43±2.04	0.82
CD44^+^	16.06±1.97	24.49±2.46	0.02

**1:**
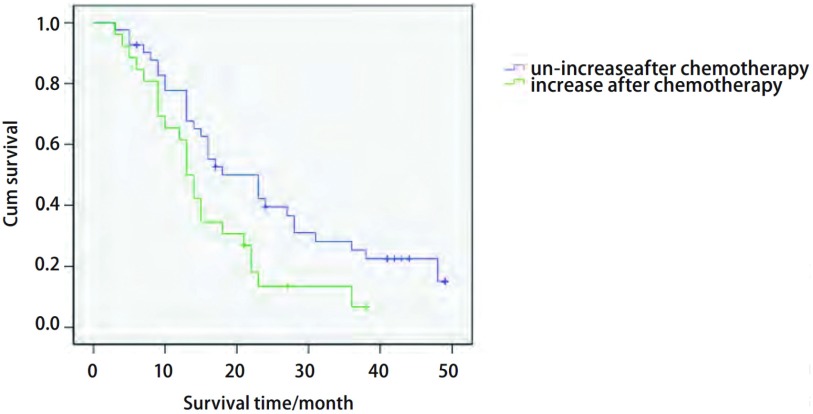
CD44^+^生存曲线显示化疗后CD44^+^表达未升高者有生存优势(*P*=0.021) Survival curve of CD44^+^ showed the patients with un-increase of CD44^+^ after chemotherapy had more survival opportunities (*P*=0.021)

## 3 讨论

一般认为具有免疫功能的淋巴细胞大致可分为T细胞、B细胞、NK细胞、巨噬细胞和树突状细胞。CD3^+^总T细胞分为CD4^+^辅助T细胞和CD8^+^细胞毒T细胞，B淋巴细胞的表面抗原有CD19和CD20，NK自然杀伤细胞的表面抗原包括CD16、CD56，调节性T细胞表面抗原有CD25(白介素2受体)，而CD44为粘附分子。

### 3.1 肺癌外周血淋巴细胞亚群的变化

在正常机体内，细胞免疫、体液免疫和固有免疫系统维持着相互间平衡的关系。肿瘤患者往往都伴有免疫功能的抑制或紊乱。许多研究^[1-4]^在肿瘤患者体内观察到CD8^+^、CD25^+^及NK细胞升高，CD4^+^、CD4^+^/CD8^+^比值及CD19^+^降低的现象，而本研究却发现CD8^+^下降，CD4^+^/CD8^+^比值、CD25^+^、NK细胞和CD19^+^上升。CD8^+^T细胞被公认为是抗肿瘤的主要效应细胞，而CD4^+^ T细胞则被认为与机体产生特异性抗肿瘤免疫有关。肺癌患者外周血中观察到CD8^+^T细胞下降的现象，可能是由于肿瘤细胞在特有的免疫逃逸机制作用下，改变了其表面特异性受体的结构，使效应性T细胞无法正确识别和结合其靶点，导致细胞毒性T细胞的增殖被反馈性抑制。而此时非特异性免疫和体液免疫所占的比例相对上升，因此NK细胞和CD19^+^细胞升高。CD4^+^T细胞较健康组虽然有所下降，但无统计学意义，故CD4^+^/CD8^+^比值相应升高。

笔者认为肿瘤患者体内淋巴细胞亚群的比例并非一成不变。Osada等^[5]^曾报道过胃癌患者外周血中CD4^+^细胞和CD8^+^细胞均下降的现象。而Patel等^[6]^总结人类乳头瘤病毒的免疫逃避机制时提出CD4^+^细胞在早期的宫颈上皮内瘤变中占主要地位，CD8^+^细胞在进展期宫颈癌中占主要地位。可见在肿瘤发生发展过程中，随着免疫微环境的改变，各淋巴细胞亚群的比例也会随之变化，需多因子联合检测才能完整反映机体的免疫状态。

本研究还比较了不同分期、不同分化程度肺癌外周血淋巴细胞亚群，发现上述8项指标均无统计学差异。NSCLC和SCLC相比CD8^+^、CD4^+^和CD4^+^/CD8^+^比值存在统计学差异。王亚娟等^[7]^曾报道CD4^+^及CD4^+^/CD8^+^比值在肺鳞癌和未分化癌间存在差异，而其它指标与分期及病理类型无相关性。这一结果与本研究结果基本相符。

### 3.2 肺癌外周血淋巴细胞亚群与化疗及预后的关系

传统观念认为肿瘤免疫以细胞免疫为主，体液免疫和固有免疫为辅。本研究证明化疗后代表细胞免疫的CD3^+^T细胞升高，而代表体液免疫和固有免疫的CD19^+^细胞、NK细胞降低。该结果需要从两方面考虑，一方面可能提示化疗对机体免疫系统具有优化作用，能活化抗肿瘤免疫应答，增强机体的抗肿瘤反应，诱使机体免疫应答朝向正向发展；另一方面化疗药物的非选择性杀伤作用，对增殖活跃的淋巴细胞也有影响^[2]^。我们采用的检测方法仅能得到各淋巴细胞的相对比例，并不能分析其绝对值，因此也可能是化疗对体液免疫和固有免疫系统的损伤作用超过了细胞免疫，使CD3^+^总T细胞的比例相对升高。可见，目前的检测方法并不能很好地评价化疗对肿瘤免疫的影响，尚待深入研究。

CD44是一种分布极为广泛的细胞表面跨膜糖蛋白，属于细胞黏附分子。其正常功能是参与淋巴细胞的激活，介导淋巴细胞的归巢、淋巴细胞向炎症部位和黏膜相关淋巴组织归位、黏附细胞外基质等。近年来发现，CD44分子在许多恶性肿瘤组织中高表达^[8, 9]^，但在外周血中的表达却并非完全一致^[10, 11]^，这可能与不同种类肿瘤的侵袭性不同有关。

本研究发现，肺癌患者外周血中CD44^+^的表达较健康人明显升高，而化疗后下降，并且在对化疗相关的淋巴细胞亚群的生存分析结果提示化疗后CD44^+^表达不升高者有生存优势(*P*=0.021)，而其他3项指标则无统计学意义，并且此作用与患者性别、年龄、生活习惯(抽烟及饮酒史)等因素无关。以上证据提示CD44^+^是肺癌的危险因素之一，其升高可能与治疗后预后不良有关，Lin等^[12]^在头颈部肿瘤研究中也有类似报道。

抗肿瘤免疫是整个免疫系统的协同作用，各种免疫细胞会随着免疫状态的改变而发生变化，联合多因子检测才能较完整的反映机体的免疫状态。化疗在杀伤肿瘤细胞的同时也引起免疫系统的改变，对于这种改变的评价还需进一步探讨。同时，本研究发现CD44粘附分子在化疗后的改变与生存时间有关，有望为临床诊疗提供一些帮助。
